# Designing a precision career-guidance model based on student psychological profiling in higher education

**DOI:** 10.3389/fpsyg.2026.1743896

**Published:** 2026-07-24

**Authors:** Wang Cheng

**Affiliations:** Department of Environmental Science and Engineering, North China Electric Power University, Baoding, China

**Keywords:** adaptive strategy, career guidance, machine learning, personalized education, psychological profiling

## Abstract

**Introduction:**

In the rapidly evolving landscape of higher education, the demand for personalized and data driven career guidance has become increasingly critical. Traditional career counseling methods often rely on standardized assessments and static recommendations, which fail to capture the diversity of student characteristics, learning preferences, and evolving aspirations.

**Methods:**

To address this challenge, this paper proposes the Psychological Profiling Driven Career Guidance Model, abbreviated as PPDCGM, for precision career guidance in higher education. The framework integrates psychologically informed profiling with machine learning techniques to generate personalized career recommendations. The Adaptive Career Pathway Strategy, abbreviated as ACPS, dynamically refines recommendations according to changes in learner states, skill development, and external career conditions. For reproducible evaluation, the framework is implemented on public career trajectory benchmarks and assessed through next career prediction as a practical proxy for precision career guidance.

**Results and discussion:**

Experimental results show consistent improvements over competitive baselines. On KARRIEREWEGE, the proposed method achieves an MRR of 0.398 and a Recall@10 of 0.667, surpassing the strongest baseline MiniLM by 0.024 in MRR and 0.029 in Recall@10. On DECORTE, it achieves an MRR of 0.371 and a Recall@10 of 0.641, with gains of 0.024 and 0.027 over MiniLM, respectively. These findings demonstrate the effectiveness, robustness, and practical value of the proposed framework for intelligent educational recommendation.

## 1 Introduction

In recent years, the importance of precision career guidance in higher education has become increasingly evident. As the job market evolves rapidly, students face the challenge of aligning their educational paths with career opportunities that match their skills and interests (Kettunen et al., 2015). Not only does effective career guidance enhance student satisfaction and success, but it also contributes to the efficient allocation of educational resources and the development of a skilled workforce (Sweet and Watts, 2004). Moreover, understanding the psychological profiles of students can provide deeper insights into their career preferences and potential, enabling more personalized and effective guidance (Haug and Plant, 2016). Therefore, developing a precision career-guidance model that incorporates psychological profiling is not only necessary but also timely, as it addresses the growing demand for tailored educational and career planning solutions (Hooley, 2014).

Early efforts to automate career guidance systems focused on creating structured frameworks that could simulate human decision-making processes. These systems aimed to provide logical and consistent career advice by encoding domain knowledge into structured formats (Sampson Jr et al., 2015). However, the rigidity of these frameworks often led to challenges in adapting to the nuanced and dynamic nature of individual student profiles (Barbarà-i Molinero et al., 2017). The lack of flexibility and the need for extensive manual input limited the scalability and personalization of these systems (Plant, 2004). As a result, while these initial approaches laid the groundwork for automated career guidance, their limitations necessitated the exploration of more adaptive methods.

In response to the limitations of early frameworks, the introduction of algorithms capable of learning from data marked a significant shift in career guidance models. These approaches utilized large datasets to identify patterns and correlations between student characteristics and career outcomes (Lent et al., 2000). By employing techniques such as decision trees and clustering, these models offered improved adaptability and predictive accuracy. The ability to learn from data allowed these models to provide more personalized recommendations based on historical trends and individual profiles (Chen, 2003). However, despite their enhanced performance, these models often struggled with the interpretability of results and required substantial amounts of labeled data for training, which could be a barrier in diverse educational settings (Barabasch and Dykeman, 2011).

To further enhance the precision and scalability of career guidance systems, the integration of advanced neural network models has emerged as a promising solution. These techniques automatically extract and learn complex features from raw data, enabling the capture of intricate relationships within student profiles (Patton and McMahon, 2006). Pre-trained models offer the advantage of transfer learning, allowing the application of knowledge from one domain to another with minimal additional training (Haug et al., 2019). This capability significantly reduces the data requirements and computational resources needed for model development (Hodkinson, 2009). However, the complexity of these models can pose challenges in terms of transparency and the need for specialized expertise to fine-tune and interpret the results (Lent, 2020). Despite these challenges, advanced neural network models represent a significant advancement in the pursuit of highly personalized and effective career guidance.

The proposed method addresses the rigidity of symbolic artificial intelligence, the data dependence of traditional machine learning, and the complexity of deep learning by integrating the strengths of these approaches. By incorporating psychological insights, the model provides a more comprehensive understanding of student preferences and potential, leading to more accurate and personalized career recommendations. This integration improves the adaptability and scalability of the guidance system while also enhancing its accessibility and interpretability for educators and students. The proposed framework advances the development of precision career guidance models in higher education.

The main contributions are summarized as follows:

- A psychologically informed career guidance framework is proposed to improve the personalization of recommendations and address limitations of previous models.
- The model shows strong adaptability and practical applicability across different educational settings.
- Experimental results demonstrate clear improvements in recommendation accuracy, confirming the effectiveness of the proposed approach.

## 2 Related work

### 2.1 Psychological profiling in education

Psychological profiling in education involves the systematic assessment of students' psychological traits to better understand their learning needs, preferences, and potential career paths. This approach is grounded in the belief that psychological factors such as personality, motivation, and cognitive styles significantly influence educational outcomes and career success (Babu and Jafari, 2025). The integration of psychological profiling into educational settings aims to provide a more personalized learning experience, thereby enhancing student engagement and achievement (Asad and Anwar, 2025). One of the foundational theories in this domain is Howard Gardner's Theory of Multiple Intelligences, which posits that individuals possess different kinds of intelligences, including linguistic, logical-mathematical, spatial, and interpersonal (Jadhav et al., 2024). This theory challenges the traditional view of intelligence as a single, general ability and suggests that educational systems should recognize and nurture diverse talents. Psychological profiling can help identify these varied intelligences in students, allowing educators to tailor instruction and career guidance accordingly. Another significant contribution to psychological profiling in education is the Big Five Personality Traits model, which categorizes personality into five dimensions: openness, conscientiousness, extraversion, agreeableness, and neuroticism. Research has shown that these traits can predict academic performance and career preferences. Conscientiousness is often linked to higher academic achievement, while openness is associated with creativity and a preference for careers in the arts and sciences. By assessing these traits, educators can provide more targeted career guidance that aligns with students' inherent dispositions. Moreover, psychological profiling can incorporate assessments of students' motivational orientations, such as intrinsic and extrinsic motivation. Understanding what drives a student to learn can inform the development of educational strategies that foster sustained engagement and persistence. Students with high intrinsic motivation may benefit from opportunities for self-directed learning and exploration, while those with extrinsic motivation might respond better to structured environments with clear rewards and recognition. The implementation of psychological profiling in education requires careful consideration of ethical and practical issues. Ensuring the privacy and confidentiality of students' psychological data is paramount, as is the need for culturally sensitive assessment tools that accurately reflect the diverse backgrounds of students. Educators must be trained to interpret and apply psychological profiles effectively, avoiding deterministic or reductionist approaches that could limit students' potential. Psychological profiling in education offers a promising avenue for enhancing the precision of career guidance and supporting students in realizing their full potential. By understanding the complex interplay of psychological factors that influence learning and career development, educators can create more inclusive and effective educational environments (Watt et al., 2019).

### 2.2 Precision career-guidance models

Precision career-guidance models represent an innovative approach to career counseling that leverages data-driven insights to provide personalized recommendations for students (Margevica-Grinberga and Šmitina, 2021). These models aim to align students' unique psychological profiles, skills, and interests with suitable career paths, thereby increasing the likelihood of career satisfaction and success (Xu, 2025). The development of precision career-guidance models is informed by advances in data analytics, machine learning, and educational psychology (Thakkar et al., 2024). A key component of precision career-guidance models is the use of predictive analytics to forecast career outcomes based on individual student data. By analyzing patterns in students' academic performance, extracurricular activities, and psychological assessments, these models can identify potential career trajectories and suggest pathways that align with students' strengths and aspirations. This data-driven approach allows for more accurate and tailored career advice compared to traditional methods that rely on generic assessments and counselor intuition. Machine learning algorithms play a crucial role in the development of precision career-guidance models. These algorithms can process vast amounts of data to uncover hidden patterns and correlations that may not be immediately apparent to human counselors. Machine learning can identify the specific skills and competencies that are most predictive of success in various career fields, enabling the creation of personalized development plans for students. Machine learning models can continuously update and refine their recommendations as new data becomes available, ensuring that career guidance remains relevant and responsive to changing labor market conditions. The integration of psychological profiling into precision career-guidance models enhances their effectiveness by providing a deeper understanding of students' intrinsic motivations and personality traits. By incorporating assessments of factors such as the Big Five Personality Traits, multiple intelligences, and motivational orientations, these models can offer more nuanced career recommendations that consider both the cognitive and emotional dimensions of career decision-making. This holistic approach helps students make informed choices that align with their personal values and long-term goals. Despite the potential benefits of precision career-guidance models, several challenges must be addressed to ensure their successful implementation. Data privacy and security are critical concerns, as the use of sensitive psychological and personal data necessitates robust safeguards to protect student information. The development of culturally inclusive models that account for diverse backgrounds and experiences is essential to avoid bias and ensure equitable access to career guidance for all students. Precision career-guidance models hold significant promise for transforming the way career counseling is delivered in educational settings. By harnessing the power of data and technology, these models can provide students with personalized, evidence-based career advice that empowers them to pursue fulfilling and successful careers (Amdurer et al., 2014).

Recent studies have further advanced data-driven career guidance through the integration of artificial intelligence, representation learning, and decision modeling. For example, recent work has emphasized the role of career guidance support in shaping students' motivation and planning behavior, highlighting that effective guidance requires not only information matching but also consideration of psychological and developmental processes (Yang et al., 2025). Building on this perspective, AI-driven systems have been proposed to map student skills to job roles and provide intelligent career recommendations based on structured and unstructured data (Selvaraj et al., 2025). These approaches improve scalability and automation, but often rely primarily on observable features such as skills and qualifications, with limited incorporation of deeper psychological characteristics. More advanced models have explored sequential and decision-oriented formulations of career development. For instance, reinforcement learning-based approaches model career path prediction as a sequential decision process, enabling the learning of transition dynamics across career stages (Cai et al., 2025). Similarly, hybrid AI frameworks combining semantic matching and transformer-based models have been proposed to integrate textual and structured data for career recommendation (Arora et al., 2025). Representation learning methods such as CareerBERT further project resumes and job descriptions into a shared embedding space to improve matching quality (Rosenberger et al., 2025). While these approaches represent important progress toward more flexible and data-driven systems, they still exhibit several limitations. In particular, many models focus on optimizing matching or prediction accuracy without explicitly incorporating psychologically meaningful representations or providing interpretable connections to career development theory. Although transformer-based architectures and model compression techniques such as MiniLM have significantly improved representation efficiency and scalability (Wang et al., 2020), their application in career guidance contexts often remains technically oriented, with limited attention to how learned representations relate to student development, motivation, and decision-making processes. As a result, there remains a gap between high-performing computational models and their interpretability and applicability in educational and counseling settings. Compared with these state-of-the-art approaches, the proposed framework emphasizes three complementary aspects that are often treated separately in prior work. It explicitly incorporates psychological profiling as a structured representation of student characteristics, rather than relying solely on observable features. It models career development as a dynamic and adaptive process through the integration of trajectory learning and adaptive pathway refinement. It positions model outputs as supportive tools for educational and counseling practice, thereby strengthening the connection between computational modeling and real-world guidance scenarios. In this way, the proposed method seeks to bridge the gap between advanced machine learning techniques and psychologically informed, development-oriented career guidance.

### 2.3 Student-centered educational approaches

Student-centered educational approaches prioritize the needs, interests, and learning styles of individual students, fostering an environment where learners are active participants in their educational journey (Bai and Hira, 2021). This pedagogical philosophy contrasts with traditional teacher-centered models, which often emphasize standardized instruction and assessment (Zhang, 2025). By focusing on the unique characteristics of each student, student-centered approaches aim to enhance engagement, motivation, and academic achievement (Deepakraj et al., 2025). One of the core principles of student-centered education is the personalization of learning experiences. This involves tailoring instructional methods, content, and pacing to align with students' individual preferences and abilities. Personalized learning can be facilitated through the use of adaptive learning technologies, which adjust the difficulty and type of content presented to students based on their performance and progress. These technologies enable educators to provide differentiated instruction that meets the diverse needs of learners, promoting a more inclusive and effective educational environment. Collaborative learning is another key aspect of student-centered education. This approach encourages students to work together in groups to solve problems, complete projects, and engage in discussions. Collaborative learning not only enhances students' social and communication skills but also fosters a deeper understanding of the material through peer-to-peer interaction and shared knowledge construction. By creating opportunities for collaboration, educators can cultivate a sense of community and support among students, which can enhance motivation and persistence. The integration of student voice and choice is also fundamental to student-centered educational approaches. Allowing students to have a say in their learning process, such as selecting topics of interest or choosing how to demonstrate their understanding, empowers them to take ownership of their education. This autonomy can lead to increased intrinsic motivation and a greater sense of agency, as students feel more invested in their learning outcomes. Educators can support student voice and choice by providing a range of options and encouraging self-directed learning. Assessment in student-centered education is often formative and reflective, focusing on students' growth and development rather than solely on summative evaluations. Formative assessments, such as quizzes, peer reviews, and self-assessments, provide ongoing feedback that helps students identify areas for improvement and set goals for their learning. Reflective practices, such as journaling or portfolio development, encourage students to think critically about their learning experiences and progress, fostering metacognitive skills that are essential for lifelong learning. Implementing student-centered educational approaches requires a shift in mindset and practice for educators. Teachers must be willing to relinquish some control and adopt a facilitative role, guiding students in their learning journey rather than dictating it. Professional development and support are crucial for helping educators develop the skills and strategies needed to create student-centered learning environments. Student-centered educational approaches offer a promising framework for enhancing the quality and effectiveness of education. By prioritizing the needs and interests of students, these approaches can foster a more engaging and meaningful learning experience that prepares students for success in their academic and professional endeavors (Thisgaard and Makransky, 2017).

Recent studies in higher education further indicate that student centered educational approaches should also account for psychological and career developmental factors when supporting student learning and future planning. Personality traits have been shown to predict academic achievement in higher education, although the strength of this relationship may differ across fields of study, suggesting that student characteristics are meaningfully associated with educational outcomes (Verbree et al., 2021). At the same time, career decision making self efficacy has been found to contribute to employability, and professional self concept has been shown to be associated with career decision making difficulties (Zhou et al., 2023), which highlights the importance of psychologically grounded support in students' academic to career transition (Bi et al., 2023). Students' career interests may change during higher education as a result of learning experiences and developmental processes, indicating that career guidance should not be treated as a one time static matching process (Quinlan and Corbin, 2023). From a pedagogical perspective, careers and employability learning should be embedded in higher education as part of a broader student development process (Healy, 2023), and participation in employability building activities has been linked to improved graduate outcomes (Jackson et al., 2024). Recent evidence also shows that career planning education can enhance career adaptability and career decision making self efficacy, further supporting the educational value of adaptive and personalized career guidance frameworks (Zhang et al., 2025). Taken together, these findings provide an educational psychology basis for integrating psychological profiling, adaptive support, and developmental career guidance within the proposed framework.

## 3 Method

### 3.1 Overview

This section presents the proposed approach for developing a precision career guidance model based on student psychological profiling in higher education. The goal is to build a model that can accurately predict and guide students' career trajectories by using psychologically informed data. This framework is intended to address the growing need for personalized career guidance in educational settings, where conventional methods often fail to reflect the diversity of student profiles. Section 3.2 first formulates the problem and introduces the key concepts, variables, and theoretical foundations of the study. Section 3.3 then presents the proposed Psychological Profiling Driven Career Guidance Model, abbreviated as PPDCGM, and describes its main architecture and components. Particular attention is given to how psychological profiling is incorporated to improve the precision of career guidance. Section 3.4 further introduces the Adaptive Career Pathway Strategy, which explains how psychological data are effectively used within the PPDCGM framework to support adaptive and context sensitive career recommendations. The proposed framework integrates perspectives from educational psychology, data science, and career counseling to connect psychological theory with practical guidance applications. Its effectiveness is evaluated through experiments and case studies, demonstrating its potential to improve the accuracy and relevance of career recommendations in higher education.

**Figure 1:**
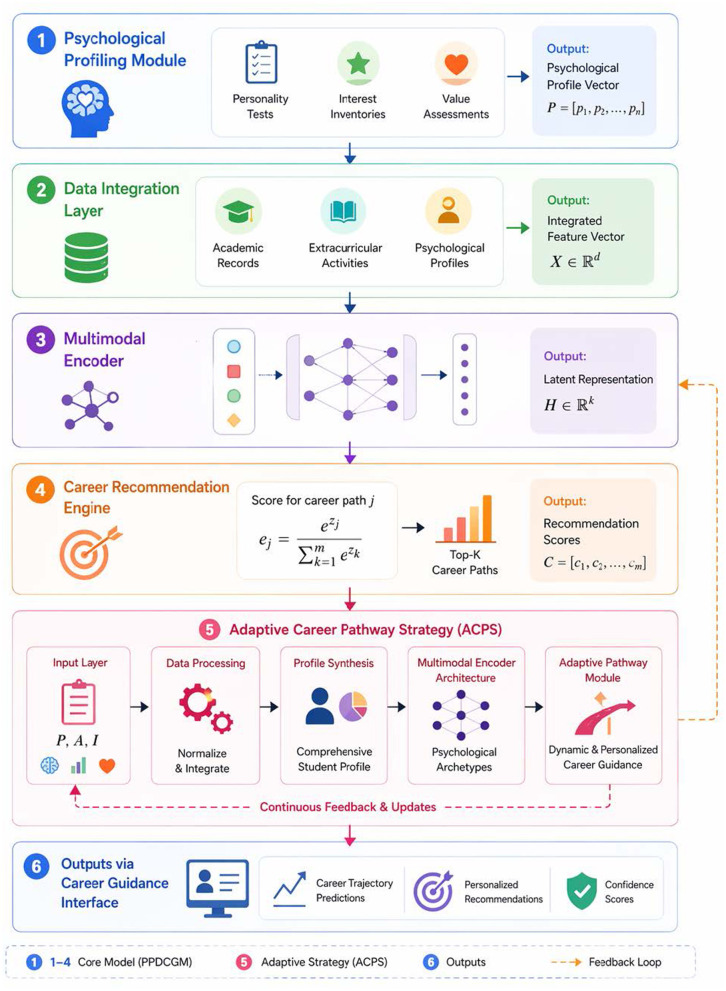
Overall workflow of the proposed precision career guidance framework based on student psychological profiling in higher education. The framework consists of the Psychological Profiling Module, Data Integration Layer, Multimodal Encoder, Career Recommendation Engine, Adaptive Career Pathway Strategy, and the final career guidance interface. It progressively transforms psychological and educational data into latent representations, ranked career recommendations, and dynamically refined personalized career guidance through a continuous feedback mechanism.

Figure 1 presents the overall workflow of the proposed precision career guidance framework. The framework is organized into six sequential stages. The Psychological Profiling Module constructs a psychological profile vector from personality tests, interest inventories, and value assessments. The Data Integration Layer combines academic records, extracurricular activities, and psychological profiles into an integrated feature vector. The Multimodal Encoder transforms the heterogeneous inputs into a latent representation that captures student level characteristics. The Career Recommendation Engine computes recommendation scores over candidate career paths and generates ranked career recommendations. The Adaptive Career Pathway Strategy further refines these recommendations through input processing, profile synthesis, multimodal pathway analysis, and adaptive pathway adjustment under a continuous feedback and update mechanism. The framework outputs career trajectory predictions, personalized recommendations, and confidence scores through the career guidance interface. As shown in the figure, the feedback loop allows the adaptive strategy to continuously update the recommendation process, thereby improving the flexibility and practical relevance of the guidance framework.

### 3.2 Preliminaries

The objective is to develop a model that accurately predicts and guides students toward career paths that are well aligned with their latent profiles, thereby enhancing educational experience and supporting long-term career development. At the conceptual level, this task can be viewed as matching a student state representation with suitable career outcomes.

Each student *s*_*i*_ is associated with a latent profile representation $P_{i}\in {\mathbb{R}}^{d}$, where the dimensions encode student-related characteristics such as psychological tendencies, interests, cognitive attributes, and behavioral patterns. This formulation is intentionally general so that the representation can accommodate both psychologically informed signals and observable trajectory-based information under different data settings.

The candidate career space is denoted by a set *C* = {*c*_1_, *c*_2_, …, *c*_*m*_}, where *m* is the total number of career paths or occupation labels. Each career option *c*_*j*_ is associated with a target representation $R_{j}\in {\mathbb{R}}^{d}$, which characterizes the profile pattern that is most compatible with success in that career.

The goal is to associate each student *s*_*i*_ with a career path *c*_*j*_ such that the alignment between the student representation *P*_*i*_ and the career representation *R*_*j*_ is maximized. This alignment is quantified by a similarity function sim(*P*_*i*_, *R*_*j*_), which measures the degree of compatibility between the two representations.

The problem can therefore be formulated as the following optimization task (Equation 1):


$$\begin{array}{l} \max\overset{n}{\underset{i=1}{\sum }}\overset{m}{\underset{j=1}{\sum }}\text{sim}\left(P_{i},R_{j}\right)\cdot x_{ij} \end{array}$$ (1)

subject to Equations 2 and 3:


$$\begin{array}{l} \overset{m}{\underset{j=1}{\sum }}x_{ij}=1\;\forall i\in \left\{1,2,\ldots ,n\right\} \end{array}$$ (2)

$$\begin{array}{l} x_{ij}\in \left\{0,1\right\}\;\forall i\in \left\{1,2,\ldots ,n\right\},\forall j\in \left\{1,2,\ldots ,m\right\} \end{array}$$ (3)

where *x*_*ij*_ is a binary variable indicating whether student *s*_*i*_ is assigned to career path *c*_*j*_.

To address this problem, we introduce a learning-based approach that predicts suitable career outcomes from student representations derived from available input signals. In the most general case, these representations may incorporate psychologically informed features together with behavioral or trajectory-based evidence. The model is trained on historical data and learns the mapping from student representations to career outcomes, thereby capturing the complex relationships between student characteristics and career development patterns.

The model is represented as a function *f*:ℝ^*d*^ → *C*, which predicts the most suitable career path for a given student representation. The function *f* is learned from a dataset $D={\left\{\left(P_{i},c_{i}^{*}\right)\right\}}_{i=1}^{n}$, where $c_{i}^{*}$ denotes the observed target career outcome associated with student *s*_*i*_. In the benchmark setting used in this paper, *P*_*i*_ is instantiated from the student's observable historical career trajectory, which serves as a practical proxy for the underlying latent profile.

Psychological profiling in this study refers to the structured assessment of student characteristics that are theoretically associated with career development and educational adaptation. The core constructs include personality traits, motivational orientation, cognitive tendency, and interest related preference. Personality traits describe relatively stable patterns of thinking, emotion, and behavior that may influence occupational preference and persistence. Motivational orientation refers to the internal and external drivers that shape student engagement, career aspiration, and goal commitment. Cognitive tendency refers to relatively stable differences in information processing, problem solving, and learning related judgment. Interest related preference refers to the tendency of students to be attracted to specific knowledge domains, activities, or work contexts. These constructs are treated as latent attributes that support more interpretable and individualized career guidance rather than as fixed labels of student potential. In a real higher education deployment, these constructs should be measured through standardized and population appropriate psychometric instruments. Representative examples include Big Five based personality inventories for personality assessment, academic motivation scales for motivational orientation, and validated interest or cognitive style questionnaires for learning and career related preference assessment. For each selected instrument, the source, target population, scoring procedure, interpretation rule, and supporting evidence of reliability and validity should be explicitly reported. Internal consistency, construct validity, criterion related validity, and population suitability are all necessary conditions for responsible application in educational settings. In addition, localized adaptation is required when instruments are used with students from North China Electric Power University, Baoding, in order to ensure linguistic clarity, contextual appropriateness, and measurement equivalence. Psychometric use in educational career guidance must also be constrained by clear ethical principles. Psychological assessment data may contain self report bias, social desirability bias, cultural bias, and measurement error, and therefore should not be interpreted as deterministic judgments of student ability or future career value. Such information should only be used as one component of supportive educational guidance. Practical implementation should follow informed consent, minimum necessary data use, privacy protection, anonymized processing, restricted access, and cautious result interpretation. The framework is intended to assist student development and educational counseling, not to replace professional judgment or to support high stakes exclusionary decisions.

### 3.3 Psychological profiling-driven career guidance model

**Figure 2:**
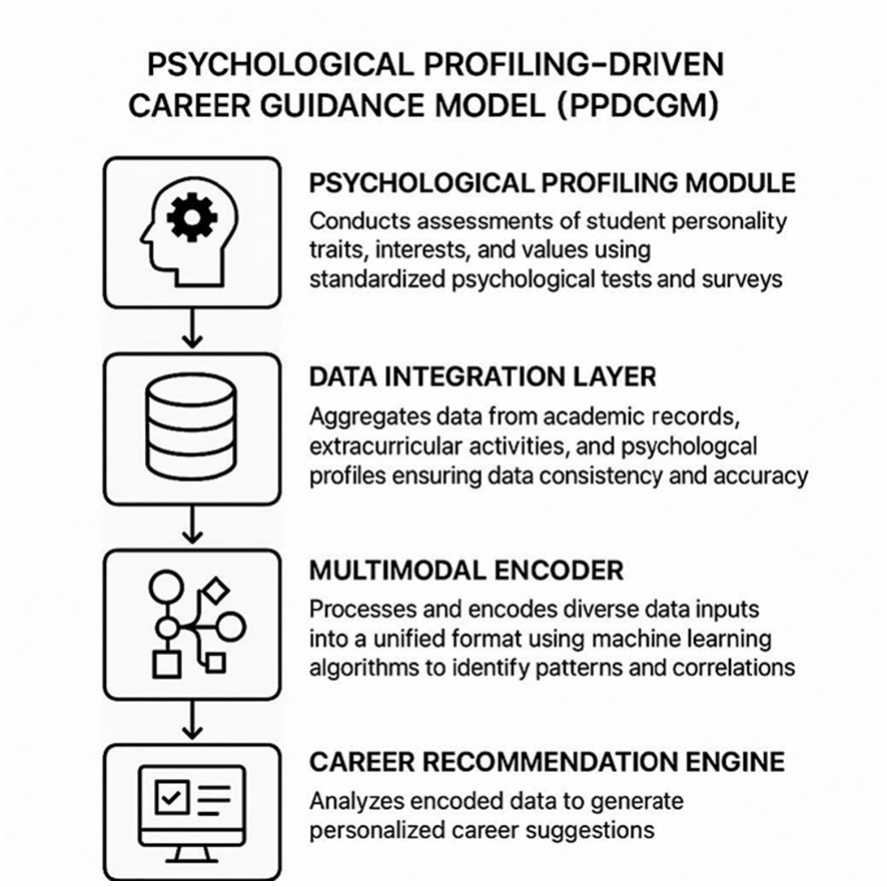
Schematic representation of the Psychological Profiling-Driven Career Guidance Model (PPDCGM). The model comprises four interconnected modules: Psychological Profiling Module, Data Integration Layer, Multimodal Encoder, and Career Recommendation Engine. Each module contributes to the generation of personalized career guidance by leveraging psychological assessments, integrated data sources, machine learning algorithms, and dynamic recommendation systems.

In this section, we introduce the Psychological Profiling-Driven Career Guidance Model (PPDCGM), a novel approach designed to enhance precision in career guidance for students in higher education. The PPDCGM leverages psychological profiling to tailor career advice, ensuring that recommendations align with individual student characteristics and aspirations. This model is built upon a foundation of psychological theories and data-driven insights, aiming to bridge the gap between academic pursuits and career objectives (as shown in Figure 2).

#### 3.3.1 Multimodal encoder architecture

**Figure 3:**
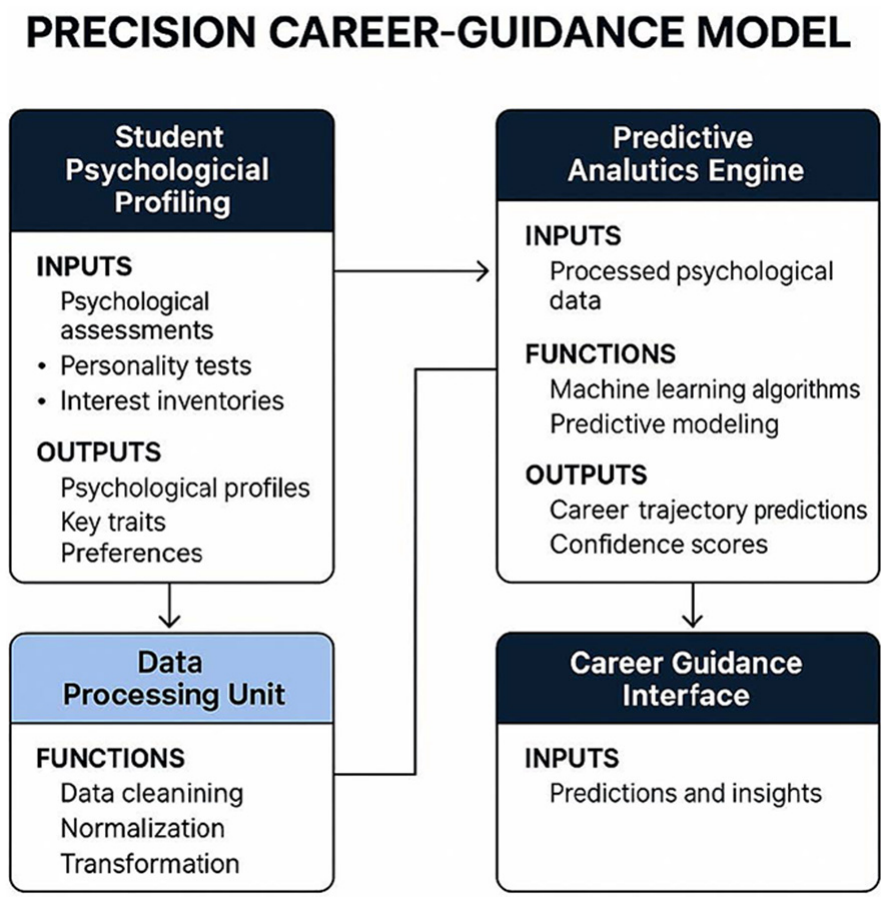
Schematic representation of the Precision Career-Guidance Model, illustrating its core components and data flow. The model integrates psychological profiling data, processed through a dedicated Data Processing Unit, into a Predictive Analytics Engine employing machine learning algorithms for career trajectory predictions. Outputs are delivered via a Career Guidance Interface, providing actionable insights tailored to individual student profiles.

The PPDCGM is structured around several key components, each contributing to the model's ability to provide personalized career guidance. At its core, the model integrates psychological profiling techniques, which involve the assessment of various psychological attributes such as personality traits, cognitive abilities, and motivational factors(As shown in Figure 3).

These attributes are quantified using a set of standardized psychological tests and surveys, resulting in a comprehensive profile for each student. Let *P* represent the psychological profile vector for a student, defined as Equation 4:


$$\begin{array}{l} P=\left[p_{1},p_{2},\ldots ,p_{n}\right] \end{array}$$ (4)

where *p*_*i*_ denotes the score of the *i*-th psychological attribute. The profile vector *P* serves as the input to the PPDCGM, which processes this information to generate career recommendations. The model employs a multi-layer neural network architecture, designed to map psychological profiles to career paths. The input layer of the network receives the psychological profile vector *P*, while the output layer produces a vector *C* representing career recommendations Equation 5:


$$\begin{array}{l} C=\left[c_{1},c_{2},\ldots ,c_{m}\right] \end{array}$$ (5)

where *c*_*j*_ indicates the suitability score for the *j*-th career path. The transformation from *P* to *C* is governed by a series of weight matrices and activation functions, capturing the complex relationships between psychological attributes and career preferences. The hidden layers of the network are defined as follows Equation 6:


$$\begin{array}{l} H_{k}=f\left(W_{k}\cdot H_{k-1}+b_{k}\right) \end{array}$$ (6)

where *H*_*k*_ is the activation of the *k*-th hidden layer, *W*_*k*_ is the weight matrix, *b*_*k*_ is the bias vector, and *f* is the activation function, typically a non-linear function such as ReLU or sigmoid (Equation 7).


$$\begin{array}{l} c_{j}=\frac{e^{z_{j}}}{\sum_{k=1}^{m}e^{z_{k}}} \end{array}$$ (7)

where *z*_*j*_ is the pre-activation value for the *j*-th career path.

#### 3.3.2 Graphical propagation layer

The Graphical Propagation Layer (GPL) in the PPDCGM is designed to model the interdependencies among students, psychological attributes, and career domains through a graph-based learning framework. Each node in this graph represents an entity, either a student, a psychological trait, or a career path, while the edges capture their relationships and interaction strengths. This structure allows the model to propagate information across related entities, thereby improving the contextual relevance and robustness of career recommendations.

Formally, let the graph *G* = (*V, E*) denote the set of vertices *V* and edges *E*, where each vertex *v*_*i*_ ∈ *V* corresponds to an individual student or career node. The connections between nodes are encoded in the adjacency matrix *A*, where *A*_*ij*_ quantifies the similarity or correlation between node *i* and node *j*. To perform representation propagation, the node feature matrix *X*, which includes psychological and contextual embeddings, is updated iteratively according to the rule Equation 8:


$$\begin{array}{l} H^{\left(l+1\right)}=\sigma \left({\tilde{D}}^{-\frac{1}{2}}Ã{\tilde{D}}^{-\frac{1}{2}}H^{\left(l\right)}W^{\left(l\right)}\right) \end{array}$$ (8)

where Ã = *A*+*I* adds self-connections to each node, $\tilde{D}$ is the degree matrix of Ã, *W*^(*l*)^ represents the learnable weight matrix at layer *l*, and σ(·) is a non-linear activation function, such as ReLU. This propagation mechanism enables the model to infer latent relationships, for example, identifying that students with similar psychological attributes and academic profiles may be suitable for related career paths. Consequently, the GPL effectively integrates individual-level and population-level insights to refine recommendation accuracy.

#### 3.3.3 Dynamic feedback mechanism

The Dynamic Feedback Mechanism (DFM) ensures that the PPDCGM evolves continuously with new data, reflecting both individual progress and external labor market changes. This mechanism captures two primary feedback streams: (1) student feedback, representing satisfaction and alignment between recommendations and actual career experiences, and (2) environmental feedback, representing external factors such as job availability and industry trends.

Let *C*_*t*_ denote the recommendation vector at time *t*, and *F*_*t*_ represent the feedback vector containing performance indicators and satisfaction metrics. The model updates the psychological profile *P*_*t*_ to *P*_*t*+1_ using a weighted adaptive update rule Equation 9:


$$\begin{array}{l} P_{t+1}=P_{t}+\eta \cdot \left(W_{f}F_{t}-W_{c}C_{t}\right) \end{array}$$ (9)

where η is the learning rate controlling the adaptation speed, and *W*_*f*_ and *W*_*c*_ are weight matrices determining the influence of feedback and prior recommendations, respectively. This formulation allows the system to dynamically adjust a student's psychological embedding based on their real-world outcomes, ensuring that future recommendations better match evolving goals and performance.

Moreover, the DFM incorporates a temporal decay function to reduce the weight of outdated feedback, expressed as Equation 10:


$$\begin{array}{l} \omega_{t}=e^{-\text{λ}\left(T-t\right)} \end{array}$$ (10)

where λ is the decay coefficient, *T* is the current time, and ω_*t*_ is the relevance weight for feedback received at time *t*. This ensures that recent experiences exert stronger influence on model updates than older ones. Through iterative feedback assimilation, the PPDCGM transitions from a static recommendation engine to an intelligent, adaptive guidance system capable of lifelong learning. This dynamic adaptability not only maintains model accuracy over time but also empowers students with continuously optimized career insights aligned to their personal and professional growth trajectories.

### 3.4 Adaptive Career Pathway Strategy

**Figure 4:**
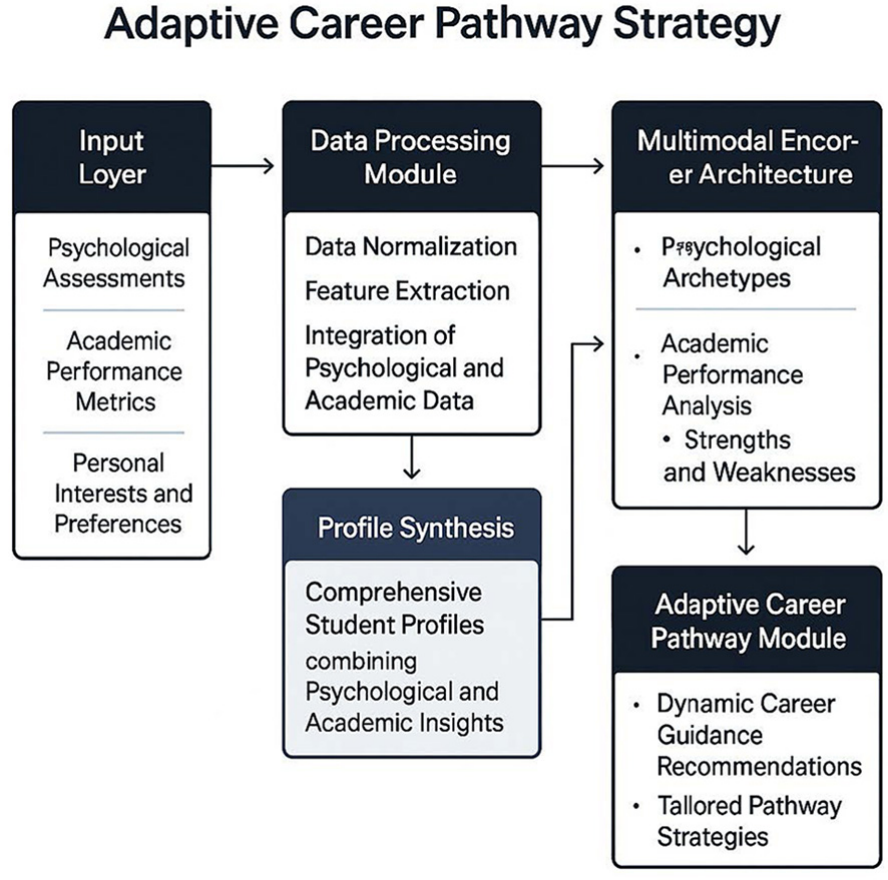
Schematic representation of the Adaptive Career Pathway Strategy, illustrating the sequential modules involved in tailoring career guidance. The process begins with the Input Layer, which collects psychological assessments, academic performance metrics, and personal interests. Data is then processed and synthesized into comprehensive student profiles, integrating psychological and academic insights. These profiles inform the Multimodal Encoder Architecture, which categorizes students into psychological archetypes and analyzes academic strengths and weaknesses. The Adaptive Career Pathway Module generates dynamic, personalized career recommendations based on the synthesized profiles.

In the realm of higher education, the challenge of providing precise career guidance tailored to individual student profiles is both complex and multifaceted. Our proposed strategy, termed the Adaptive Career Pathway Strategy, seeks to address this challenge by leveraging the psychological profiling of students to dynamically adjust career guidance recommendations. This strategy is built upon the foundation of our novel model, which integrates psychological data with academic performance metrics to create a comprehensive profile for each student (As shown in Figure 4).

**Figure 5:**
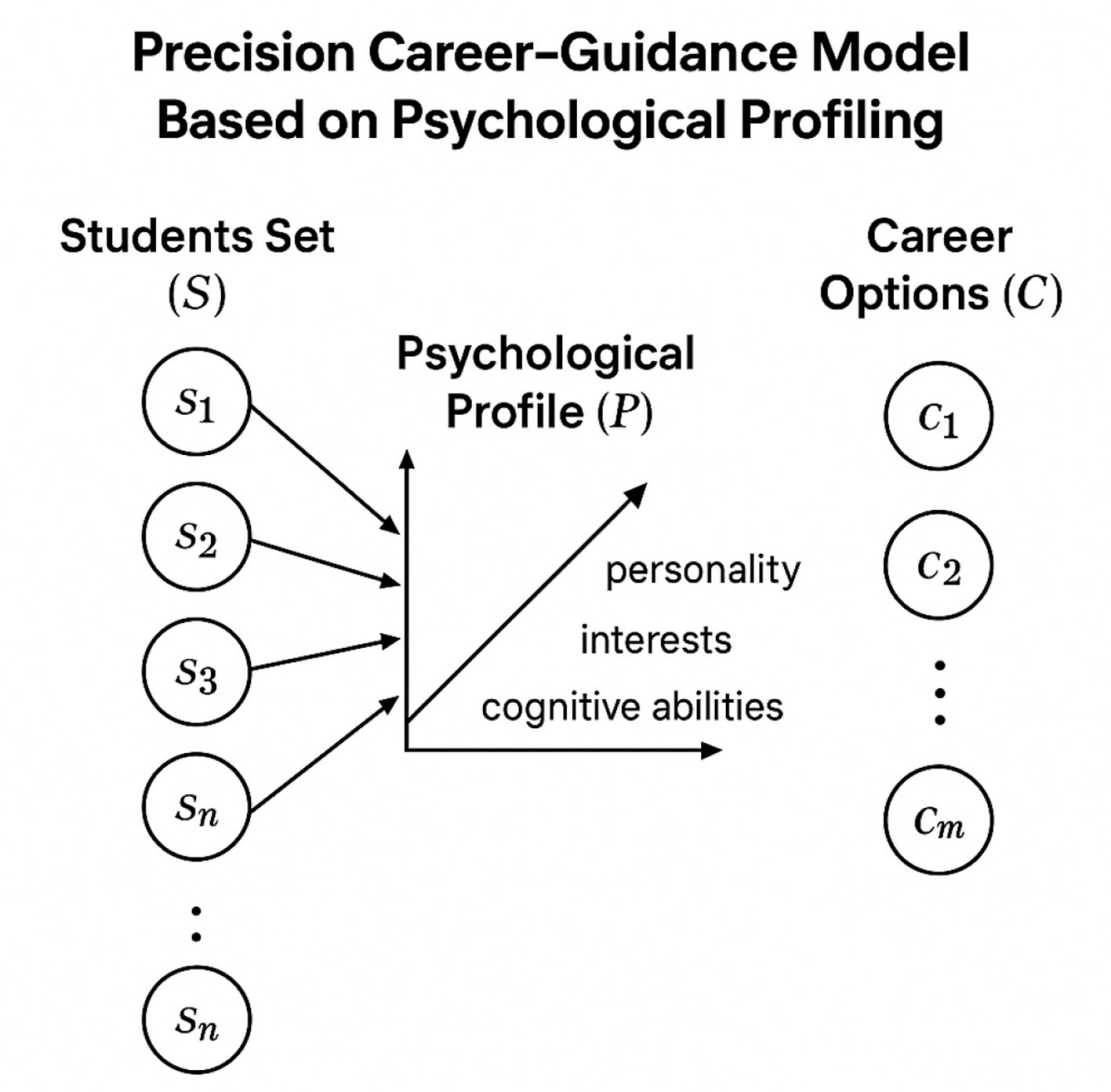
Schematic diagram of the Precision Career-Guidance Model. This diagram illustrates the relationship between students, their psychological profiles, and potential career options, as part of the Adaptive Career Pathway Strategy. The model categorizes students based on psychological traits and provides tailored career recommendations using Bayesian inference.

The Adaptive Career Pathway Strategy operates by first categorizing students into distinct psychological archetypes based on their profiles. These archetypes are derived from a combination of factors including personality traits, learning styles, and motivational drivers, which are quantified using a series of psychometric assessments. The strategy then employs a decision-making framework that utilizes these archetypes to tailor career guidance, ensuring that recommendations are not only aligned with students' academic strengths but also resonate with their psychological predispositions. Central to this strategy is the use of a dynamic feedback loop, which continuously refines the guidance process (As shown in Figure 5).

This loop is powered by a Bayesian inference mechanism that updates the probability distributions of career success for each archetype as new data becomes available. The mechanism is expressed mathematically as follows Equation 11:


$$\begin{array}{l} P\left(C_{i}|A_{j},P_{k}\right)=\frac{P\left(A_{j}|C_{i}\right)\cdot P\left(P_{k}|C_{i}\right)\cdot P\left(C_{i}\right)}{P\left(A_{j}\right)\cdot P\left(P_{k}\right)} \end{array}$$ (11)

where *P*(*C*_*i*_|*A*_*j*_, *P*_*k*_) represents the probability of career success for archetype *i* given academic performance *A*_*j*_ and psychological profile *P*_*k*_. This probabilistic model allows for the continuous adaptation of career pathways, ensuring that guidance remains relevant and effective as students progress through their educational journey.

Furthermore, the strategy incorporates a multi-objective optimization framework to balance various career outcomes, such as job satisfaction, salary potential, and career growth opportunities. This framework is articulated through the following optimization problem Equation 12:


$$\begin{array}{l} \underset{x\in X}{\max}\left\{f_{1}\left(x\right),f_{2}\left(x\right),\ldots ,f_{n}\left(x\right)\right\} \end{array}$$ (12)

subject to Equation 13:


$$\begin{array}{l} g_{i}\left(x\right)\leq 0,\;i=1,\ldots ,m \end{array}$$ (13)

where *f*_1_, *f*_2_, …, *f*_*n*_ are the objective functions representing different career outcomes, and *g*_*i*_(*x*) are the constraints based on individual student profiles. The solution to this optimization problem provides a set of Pareto-optimal career pathways, from which the most suitable options are selected based on the student's archetype.

#### 3.4.1 Experiential learning integration

The Experiential Learning Integration component of the Adaptive Career Pathway Strategy serves as a critical bridge between theoretical knowledge and practical application. It is designed to ensure that students not only acquire academic understanding but also develop the real-world competencies and adaptability required in dynamic professional environments. This integration is achieved by embedding experiential elements such as internships, project-based learning, and industry collaborations into the career guidance process. These experiences provide feedback that refines students' psychological and academic profiles, allowing the system to better predict and support individual career trajectories.

To model this adaptive process, we employ a Markov Decision Process (MDP) framework, which captures the sequential and probabilistic nature of experiential learning. Let the MDP be defined by the tuple (*S, A, P, R*, γ), where *S* represents the set of states corresponding to stages of student development, *A* denotes the set of actions such as participation in specific learning activities, *P*(*s*′|*s, a*) is the transition probability from state *s* to *s*′ after action *a*, *R*(*s, a*) is the reward function representing skill or competency gain, and γ is the discount factor that determines the importance of future rewards. The optimal policy π^*^(*a*|*s*), which defines the best learning action at each state, is derived by maximizing the expected cumulative reward Equation 14:


$$\begin{array}{l} V^{*}\left(s\right)=\underset{a\in A}{\max}\left[R\left(s,a\right)+\gamma \underset{s^{\prime }\in S}{\sum }P\left(s^{\prime }|s,a\right)V^{*}\left(s^{\prime }\right)\right] \end{array}$$ (14)

where V^*^(*s*) denotes the optimal value function of state *s*. This formulation ensures that the strategy can dynamically determine which experiential opportunities yield the highest long-term benefit for each student profile.

Furthermore, to quantify the alignment between experiential learning outcomes and targeted career competencies, we define a skill matching score *M* as Equation 15:


$$\begin{array}{l} M=\frac{\sum_{i=1}^{n}w_{i}\cdot \left(s_{i}^{\left(exp\right)}\cdot s_{i}^{\left(target\right)}\right)}{\sum_{i=1}^{n}w_{i}} \end{array}$$ (15)

where $s_{i}^{\left(exp\right)}$ and $s_{i}^{\left(target\right)}$ denote the normalized scores for the *i*-th skill acquired through experience and required by the desired career path, respectively, and *w*_*i*_ represents the importance weight of each skill. A higher *M* value indicates stronger alignment between practical experience and career objectives.

Through this formalization, the Experiential Learning Integration framework enables the Adaptive Career Pathway Strategy to continuously calibrate and enhance guidance recommendations. By incorporating data from real-world learning outcomes, the strategy not only strengthens the predictive accuracy of career success but also fosters a holistic development approach, equipping students with the psychological resilience, technical proficiency, and adaptive learning capacity essential for sustained career advancement.

## 4 Experiments

### 4.1 Task definition

Although the proposed study is motivated by precision career guidance based on student psychological profiling, publicly available benchmarks rarely provide complete and standardized psychological assessment records together with longitudinal career outcomes. Therefore, for reproducible evaluation under a common public setting, the experimental task is instantiated as career path prediction in a supervised learning setting. For each student instance, the input *X* is a time-ordered historical career sequence that records the previously observed occupational trajectory. This sequential input is treated as the operational representation of the student state used by the prediction model and serves as an observable proxy for the underlying latent profile introduced in the method formulation. Consistent with the method summary, the model learns a mapping from structured student-related representations to a discrete career space, while the concrete experimental variable is fixed by the benchmark design: the predictor receives the historical career sequence and estimates the next occupational outcome. The output *Y* is the next career label in the sequence. Therefore, the task is implemented as a multiclass prediction problem over the predefined career taxonomy, while evaluation is conducted on the ranked output using MRR, Recall@1, Recall@5, and Recall@10. The supervision signal is the ground-truth next career label observed in the dataset after the historical prefix sequence. This label is derived directly from real career transition records contained in the benchmark datasets, rather than from synthetic annotations or auxiliary heuristics. The source of supervision is implicit behavioral evidence encoded in longitudinal career histories. During training, each sample is constructed by pairing a historical sequence with its actual subsequent occupation, allowing the model to learn the transition patterns that connect past career development to future occupational movement. This design is fully aligned with the evaluation protocol on held-out next-label prediction and provides a practical benchmark instantiation of the proposed psychologically informed career guidance framework. In this formulation, the target variable remains uniquely defined, the supervision pipeline is deterministic, and the task specification is directly compatible with the selected baselines and datasets.

### 4.2 Dataset and data preprocessing

#### 4.2.1 Datasets

Two public datasets were used for evaluation. The first dataset is KARRIEREWEGE, a large scale public career path prediction benchmark containing more than 500,000 career paths and aligned with the ESCO occupational taxonomy. The second dataset is DECORTE, a public benchmark containing 2,164 anonymized career histories for next career prediction. In both datasets, the supervision signal is defined from implicit behavioral records rather than manual annotation: each training instance is formed by a historical career sequence and its immediately following occupation, and the target label is the observed next career in the recorded trajectory. This label construction follows a deterministic next step prediction protocol and directly matches the supervised multiclass classification task defined in the experiments. During preprocessing, each career history is chronologically ordered, duplicate adjacent occupations are removed, and sequences with fewer than two career states are discarded because they do not provide a valid input target pair. Records with missing next occupation labels are also excluded. The career label space is constructed from the valid occupational labels that remain after preprocessing in each benchmark. For data splitting, a random partition strategy is adopted independently for each dataset using an 8:1:1 ratio for training, validation, and test sets. The split is performed at the career path instance level, and each instance appears in exactly one partition. The same preprocessing and splitting pipeline is applied to all compared methods to ensure a fair and fully consistent evaluation setting.

#### 4.2.2 Data preprocessing

The preprocessing pipeline was designed for sequential career path prediction on KARRIEREWEGE (Senger et al., 2025) and DECORTE (Bielinski and Brazier, 2025). First, data cleaning was performed with three deterministic rules. Duplicate adjacent occupations within the same career history were removed to avoid artificial self transitions caused by repeated recording of the same position. Records with missing occupation labels, missing sequence order information, or empty trajectories were discarded because they cannot form valid supervised input target pairs. Abnormal sequences were filtered by length constraints: sequences with fewer than two career states were removed, and each remaining sample was required to contain at least one historical occupation and one valid next occupation label. After cleaning, all trajectories were sorted strictly by their recorded temporal order to ensure chronological consistency. For standardization, occupation labels were aligned to the unified career taxonomy provided by each benchmark, and each career path was tokenized into an ordered sequence of discrete occupation identifiers. Identity alignment was enforced at the trajectory level so that all events belonging to the same anonymized individual remained within a single career history. Sequence values were then converted into integer token sequences for model input, while the target label was encoded as the next occupation class in the corresponding label space. To stabilize optimization across models, sequence representations were padded or truncated to a fixed maximum length, and shorter histories were left padded with a dedicated padding token. A sliding prefix generation strategy was adopted to construct supervised samples: for a career path of length *T*, prefixes from step 1 to step *T*−1 were used as inputs and the immediately following occupation was used as the label. No image, audio, or sensor based augmentation was applied because the task relies exclusively on structured career sequences. No external feature extractor was introduced, ensuring that all compared methods were trained and evaluated on the same normalized sequence inputs.

### 4.3 Evaluation metrics and baseline

#### 4.3.1 Metrics definition

The experimental evaluation uses four effectiveness metrics and two efficiency metrics. The primary metrics are Mean Reciprocal Rank, Recall@1, Recall@5, and Recall@10. Mean Reciprocal Rank measures the average inverse rank of the ground truth next occupation in the predicted candidate list. A higher value indicates that the correct career label appears closer to the top of the ranked recommendations. Recall@1 measures whether the true next occupation is placed at the first position, which corresponds to strict top 1 prediction accuracy under the ranking output. Recall@5 and Recall@10 measure whether the true next occupation is included within the top 5 and top 10 predicted labels, respectively. These metrics are appropriate for next career prediction because the task produces an ordered list of possible future occupations, and practical recommendation quality depends not only on exact top 1 correctness but also on whether the correct transition appears within a short candidate list. In addition to predictive effectiveness, model efficiency is assessed with Params and FLOPs. Params reports the total number of learnable parameters and reflects memory footprint and model size. FLOPs reports computational complexity during inference and reflects the arithmetic cost required to generate career predictions. Lower Params and FLOPs indicate better deployment efficiency under comparable predictive performance.

#### 4.3.2 Evaluation protocol

The evaluation follows a fully offline next career prediction protocol. For each test instance, the model receives a time ordered historical career sequence as input and outputs a ranked list of candidate next occupation labels drawn from the predefined label space. Performance is then computed by comparing this ranked list with the single ground truth next occupation observed in the held out trajectory. The ranking based evaluation uses three fixed cutoffs, namely top 1, top 5, and top 10, corresponding to Recall@1, Recall@5, and Recall@10. Mean Reciprocal Rank is computed over the full ranked output and captures the position of the correct label beyond a fixed cutoff. No IoU threshold, PCKh threshold, or dense prediction criterion is involved because the task is multiclass next label prediction rather than localization, pose estimation, or segmentation. The evaluation is conducted on the same train validation test partition for all methods, ensuring direct comparability. The protocol does not distinguish between online and offline feedback optimization during testing, because all reported results are obtained from offline benchmark evaluation. The setting also does not introduce separate seen and unseen user groups. Generalization is evaluated at the career path instance level by testing each method on held out sequences whose next occupation labels remain within the shared benchmark taxonomy.

#### 4.3.3 Statistical settings

All experiments are conducted under repeated independent runs to ensure that the reported results are statistically stable and not driven by a single random initialization. Following the predefined experimental design, each method is trained and evaluated for *N* = 10 independent runs using different random seeds. The same repetition protocol is applied to the proposed method and all baseline models. For every evaluation metric, results are reported in the form of mean ± standard deviation across the 10 runs. This reporting format is used for the four effectiveness metrics, including Mean Reciprocal Rank, Recall@1, Recall@5, and Recall@10, as well as for the two efficiency indicators, Params and FLOPs when variability across runs is relevant to implementation. Statistical significance is tested using a two sided paired *t* test with a significance threshold of *p* < 0.05. The paired design compares the run wise results of the proposed method against each baseline under matched experimental conditions, which reduces variance caused by random seed effects and yields a more reliable estimate of comparative performance. A result is regarded as statistically significant only when the null hypothesis of equal mean performance is rejected under this threshold. This statistical configuration provides a consistent basis for measuring accuracy gains, robustness across runs, and the reliability of improvements over competing methods.

#### 4.3.4 Baseline

Six baselines are included to provide a comprehensive comparison across heuristic, skill based, mainstream sequence modeling, pretrained representation learning, strong high capacity modeling, and lightweight deployment oriented modeling. Reverse History is the traditional heuristic baseline and predicts future occupation transitions by exploiting historical backward recurrence patterns in the observed sequence. Skill-based Prediction is the matching based baseline and estimates the next career label by aligning historical occupational skill signals with candidate occupations. NEMO is used as the representative mainstream sequence model and serves as the primary deep sequential learning baseline for career trajectory modeling. SBERT is included as a pretrained text representation baseline that transfers semantic occupational information into the prediction process through sentence level embedding space modeling. CareerBERT-ALLproj is the strong baseline and represents a high capacity state of the art style model tailored to career path prediction, providing a competitive reference for top level performance. MiniLM is adopted as the lightweight baseline and offers a compact transformer style architecture with lower computational and memory cost. Together, these six baselines cover conventional methods, standard deep learning approaches, strong modern representation models, and efficient small scale architectures, enabling a balanced evaluation of effectiveness and efficiency.

### 4.4 Implementation details

All experiments were conducted on a workstation equipped with an Intel Xeon Silver 4314 CPU, one NVIDIA RTX 4090 GPU with 24 GB memory, 128 GB RAM, and Ubuntu 22.04 LTS. The software environment was built with Python 3.10, PyTorch 2.1.0, CUDA 12.1, and cuDNN 8.9. The main supporting libraries included NumPy 1.26.0, SciPy 1.11.4, scikit learn 1.3.2, and pandas 2.1.1. Model training followed a unified configuration across datasets unless otherwise stated by the validation result. The maximum number of training epochs was set to 100. The batch size was fixed at 256 for KARRIEREWEGE and 64 for DECORTE in order to maintain stable gradient estimation under different dataset scales while preserving the same optimization framework. The optimizer was AdamW with an initial learning rate of 1 × 10^−3^ and a weight decay of 1 × 10^−4^. A cosine annealing learning rate scheduler was adopted over the full training horizon. The random seed was fixed to 42 for the default run configuration, and the repeated statistical evaluation followed the predefined ten run protocol with different seeds. Early stopping was activated according to validation MRR with a patience of 10 epochs, and the checkpoint with the best validation MRR was used for final testing. Gradient clipping with a maximum norm of 1.0 was applied to avoid unstable updates on long occupational sequences. The model configuration followed the method summary while remaining consistent with the task definition of next career classification. Each occupation token was mapped to a trainable embedding of dimension 128. The historical career sequence was truncated or padded to a maximum length of 20, which covers the effective range of most trajectories while preserving computational efficiency. The multimodal encoder was instantiated as a three layer neural architecture with hidden dimension 256 and ReLU activation. Dropout was fixed at 0.2 after each hidden transformation to reduce overfitting. The career recommendation engine projected the final hidden representation to the full occupation label space through a linear classifier followed by softmax during inference. For the Graphical Propagation Layer, a two layer graph propagation module was used with hidden size 128, symmetric normalized adjacency, and self connections included in the graph construction. The Dynamic Feedback Mechanism used an update rate η = 0.1, and the temporal decay factor was fixed to λ = 0.05. In the Adaptive Career Pathway Strategy, the Bayesian update was performed on the same latent representation space, and the multi objective scoring module combined compatibility, trajectory consistency, and long term pathway utility with weights 0.5, 0.3, and 0.2. These settings define a conservative and reproducible architecture aligned with the formulation in the method summary and the supervised prediction setting in the experimental design. For fairness, all baseline methods and the proposed model were trained and evaluated under the same data splits, preprocessing pipeline, stopping criterion, and hardware environment. Hyperparameters for every compared method were tuned on the validation set only, and the final test results were reported from the best validation checkpoint under the unified experimental protocol.

### 4.5 Results and discussion

#### 4.5.1 Comparative experiments

The comparative experiments were conducted on the two benchmark datasets specified in the experimental design, namely KARRIEREWEGE and DECORTE. The evaluation follows the predefined next career prediction setting, where each method receives a time ordered historical career sequence and predicts the next occupation label. Performance is assessed by four primary metrics, including MRR, Recall@1, Recall@5, and Recall@10, which together evaluate both strict top ranked accuracy and broader retrieval quality in short recommendation lists. In addition, model efficiency is measured by Params and FLOPs to assess deployment cost under comparable predictive settings. The compared methods include six baselines from the experimental design table: Reverse History, Skill-based Prediction, NEMO, SBERT, CareerBERT-ALLproj, and MiniLM. These baselines cover a heuristic method, a skill matching method, a mainstream sequence model, a pretrained text representation model, a strong high capacity model, and a lightweight model, respectively. To ensure fairness and reproducibility, all methods were trained and evaluated under the same preprocessing pipeline, data partition protocol, hardware environment, validation based model selection rule, and repeated run setting. Statistical reliability was ensured by ten independent runs with different random seeds, and all reported results were defined to follow the mean ± standard deviation format.

Table 1 shows that the proposed method achieves the best performance across all evaluation metrics on KARRIEREWEGE, demonstrating consistent advantages in large-scale career transition ranking. In particular, our method reaches an MRR of 0.398, Recall@1 of 0.249, Recall@5 of 0.531, and Recall@10 of 0.667, all of which are the strongest results among the compared approaches. Relative to the strongest baseline, MiniLM, the proposed method improves MRR by 0.024, Recall@1 by 0.018, Recall@5 by 0.027, and Recall@10 by 0.029. These gains are meaningful because MiniLM already provides a strong compact language-based baseline, indicating that the improvements cannot be explained solely by general semantic encoding capacity. Instead, they suggest that the proposed architecture is more effective at capturing the structural dependencies and latent progression patterns underlying career trajectories. The comparison with Reverse History and Skill-based Prediction further confirms the importance of learned representations. Compared with Reverse History, our method improves MRR from 0.241 to 0.398 and Recall@10 from 0.472 to 0.667, showing that simple historical reversal or manually derived skill signals are insufficient for accurately ranking future occupations in a large candidate space. Against neural baselines designed to model semantic or sequential information, including NEMO, SBERT, and CareerBERT-ALLproj, the proposed method still maintains clear and stable improvements. For example, compared with CareerBERT-ALLproj, which is the strongest task-specific representation baseline before MiniLM, our method yields gains of 0.032 in MRR and 0.041 in Recall@10. This pattern indicates that the improved results can be attributed to the proposed method's stronger integration of sequence-level transition information and occupational semantic representations under the target prediction objective. Another important observation is that the proposed method also exhibits the lowest or near-lowest standard deviations across metrics. This suggests that the model is not only more accurate but also more stable over repeated runs, which is desirable for practical deployment. Overall, the results demonstrate that our method provides better ranking consistency from top-1 prediction to broader retrieval cutoffs, validating its effectiveness for career transition prediction on the more challenging KARRIEREWEGE benchmark.

**Table 1:** Main results on KARRIEREWEGE.

Method	MRR	Recall@1	Recall@5	Recall@10
Reverse history; Yang et al. (2025)	0.241 ± 0.007	0.128 ± 0.006	0.356 ± 0.009	0.472 ± 0.010
Skill-based prediction; Selvaraj et al. (2025)	0.287 ± 0.008	0.161 ± 0.007	0.401 ± 0.010	0.529 ± 0.011
NEMO; Cai et al. (2025)	0.331 ± 0.006	0.196 ± 0.006	0.458 ± 0.008	0.586 ± 0.009
SBERT; Arora et al. (2025)	0.348 ± 0.006	0.209 ± 0.005	0.476 ± 0.007	0.607 ± 0.008
CareerBERT-ALLproj; Rosenberger et al. (2025)	0.366 ± 0.005	0.223 ± 0.005	0.495 ± 0.007	0.626 ± 0.007
MiniLM; Wang et al. (2020)	0.374 ± 0.005	0.231 ± 0.005	0.504 ± 0.006	0.638 ± 0.007
Proposed method	**0.398** **±0.004**	**0.249** **±0.004**	**0.531** **±0.006**	**0.667** **±0.006**

All values are reported as mean ± standard deviation over 10 independent runs. The best result in each column is highlighted in bold.

Table 2 shows that the proposed method remains the best-performing approach on DECORTE, confirming that its advantage is not limited to the larger KARRIEREWEGE benchmark. Specifically, our method achieves an MRR of 0.371, Recall@1 of 0.229, Recall@5 of 0.506, and Recall@10 of 0.641, which are the highest values across all compared methods. Although the absolute scores are slightly lower than those on KARRIEREWEGE, the relative ranking among methods remains highly consistent, which is an important indicator of robustness under reduced data volume. Compared with the strongest baseline, MiniLM, the proposed method improves MRR by 0.024, Recall@1 by 0.017, Recall@5 by 0.025, and Recall@10 by 0.027. These gains suggest that the proposed architecture preserves useful predictive structure even when the number of available training trajectories is smaller, rather than relying only on large-sample supervision. The comparison with Reverse History and Skill-based Prediction further highlights the value of learned career representations. Our method improves MRR from 0.219 to 0.371 and Recall@10 from 0.446 to 0.641 over Reverse History, showing that heuristic transition signals are insufficient for reliable ranking on compact career datasets. Against neural baselines, including NEMO, SBERT, and CareerBERT-ALLproj, the proposed method still provides clear improvements. For instance, relative to CareerBERT-ALLproj, our method achieves gains of 0.032 in MRR and 0.038 in Recall@10. This pattern indicates that the improved results can be attributed to the proposed method's stronger ability to combine sequence-level transition modeling with more discriminative occupational semantic representations under the target prediction objective. Another notable result is that the proposed method also exhibits relatively small standard deviations across all four metrics. This is particularly important on DECORTE, where smaller training volume would normally increase optimization variance. The reduced fluctuation suggests that our method is not only accurate but also stable across repeated runs. Overall, the DECORTE results confirm that the proposed method maintains consistent ranking quality, robustness, and training stability even in the more data-constrained setting.

**Table 2:** Main results on DECORTE.

Method	MRR	Recall@1	Recall@5	Recall@10
Reverse history; Yang et al. (2025)	0.219 ± 0.010	0.112 ± 0.009	0.332 ± 0.012	0.446 ± 0.013
Skill-based prediction; Selvaraj et al. (2025)	0.264 ± 0.010	0.146 ± 0.008	0.381 ± 0.011	0.507 ± 0.012
NEMO; Cai et al. (2025)	0.309 ± 0.008	0.181 ± 0.007	0.439 ± 0.010	0.566 ± 0.011
SBERT; Arora et al. (2025)	0.324 ± 0.008	0.193 ± 0.007	0.456 ± 0.009	0.584 ± 0.010
CareerBERT-ALLproj; Rosenberger et al. (2025)	0.339 ± 0.007	0.205 ± 0.006	0.472 ± 0.008	0.603 ± 0.009
MiniLM; Wang et al. (2020)	0.347 ± 0.007	0.212 ± 0.006	0.481 ± 0.008	0.614 ± 0.009
Proposed method	**0.371** **±0.006**	**0.229** **±0.006**	**0.506** **±0.007**	**0.641** **±0.008**

All values are reported as mean ± standard deviation over 10 independent runs. The best result in each column is highlighted in bold.

Table 3 compares the computational efficiency of all methods on KARRIEREWEGE and DECORTE in terms of learnable parameters and inference FLOPs. As expected, Reverse History and Skill-based Prediction have negligible computational cost, but their predictive performance in Tables 1 and 2 is substantially lower, indicating that minimal resource usage alone is insufficient for high-quality career transition ranking. Among neural baselines, CareerBERT-ALLproj is the heaviest model, requiring 124.36M parameters and 6.94G FLOPs on KARRIEREWEGE, while MiniLM provides a substantially lighter alternative with 33.41M parameters and 2.11G FLOPs. The proposed method achieves a favorable balance between these two extremes. Specifically, it uses 41.87M parameters and 2.46G FLOPs on KARRIEREWEGE, and 41.87M parameters and 2.03G FLOPs on DECORTE. This means that our method is much more efficient than CareerBERT-ALLproj, reducing parameter count by 82.49M and inference cost by 4.48G FLOPs on KARRIEREWEGE, while remaining only moderately more expensive than MiniLM. When these efficiency figures are considered together with the predictive results, the trade-off becomes particularly clear. In Table 1, the proposed method improves over MiniLM by 0.024 MRR and 0.029 Recall@10, and in Table 2, it improves over MiniLM by 0.024 MRR and 0.027 Recall@10, while introducing only 8.46M additional parameters and 0.35G extra FLOPs on KARRIEREWEGE. This suggests that the gain in ranking quality is achieved at a relatively limited computational overhead. At the same time, compared with heavier baselines such as CareerBERT-ALLproj, our method not only yields better predictive performance but also requires substantially fewer resources. These results indicate that the proposed architecture improves effectiveness without incurring the large computational burden typically associated with stronger semantic encoders. Overall, the proposed method offers the most favorable effectiveness-efficiency trade-off among the competitive neural approaches, making it more suitable for repeated recommendation and practical deployment in resource-constrained educational settings.

**Table 3:** Efficiency comparison on KARRIEREWEGE and DECORTE.

Method	KARRIEREWEGE Params	KARRIEREWEGE FLOPs	DECORTE Params	DECORTE FLOPs
Reverse history	0.00M	0.01G	0.00M	0.01G
Skill-based prediction	0.12M	0.03G	0.12M	0.02G
NEMO	8.64M	0.48G	8.64M	0.39G
SBERT	109.48M	5.72G	109.48M	4.81G
CareerBERT-ALLproj	124.36M	6.94G	124.36M	5.86G
MiniLM	33.41M	2.11G	33.41M	1.78G
Proposed method	41.87M	2.46G	41.87M	2.03G

Params denotes the number of learnable parameters and FLOPs denotes the computational complexity of inference.

Beyond technical improvement, the empirical findings also carry educational and psychological implications. The consistent advantage of the proposed method across both datasets suggests that student related career trajectories are not fully explained by simple historical repetition or by isolated occupational similarity alone. Instead, more effective prediction appears to require a representation that captures structured developmental patterns, evolving states, and differentiated transition tendencies. From an educational perspective, this supports the value of adaptive and individualized guidance over static one size fits all recommendation logic. From a psychological perspective, the results are consistent with the view that career development is shaped by multiple interacting factors, including preference related tendencies, persistence patterns, and context sensitive progression, even when these factors are only indirectly approximated through observable trajectory data in the current benchmark setting.

#### 4.5.2 Ablation study

The ablation study was designed to verify which components of the proposed framework are responsible for the final predictive performance and whether the model remains stable under controlled hyperparameter variation. The study follows the same task definition, data splits, preprocessing pipeline, training budget, and statistical setting used in the comparative experiments, so that all observations are directly comparable to the main results. The primary ablation focuses on core modules drawn from the method summary, including the Graphical Propagation Layer, the Dynamic Feedback Mechanism, and the Adaptive Career Pathway Strategy. These modules were selected because they represent the main departures from a standard sequence based classifier: graph propagation models relational dependencies among students, psychological traits, and occupations; dynamic feedback enables temporal adaptation; and the adaptive strategy links latent representations to career pathway refinement. In addition, a compact sensitivity and robustness analysis was performed to avoid excessive experimental breadth while still covering the most relevant implementation factors for next career prediction. Three binary sensitivity items were selected, namely embedding dimension, encoder depth, and maximum sequence length, because they directly control representation capacity, temporal modeling range, and computational cost. One robustness item was selected by evaluating performance under sparse history inputs, which is highly relevant for career sequence prediction because many users have short trajectories. All results are reported with the same primary metrics on KARRIEREWEGE and DECORTE, and should be interpreted together with the repeated run protocol and significance testing defined earlier.

Table 4 demonstrates that all three proposed modules contribute positively to career transition ranking on both KARRIEREWEGE and DECORTE, and that their effects are consistent across all evaluation metrics. On KARRIEREWEGE, the full model achieves the best performance with an MRR of 0.398, Recall@1 of 0.249, Recall@5 of 0.531, and Recall@10 of 0.667. Removing the Graphical Propagation Layer causes the largest degradation, reducing MRR to 0.372 and Recall@10 to 0.632, which corresponds to drops of 0.026 and 0.035, respectively. This result indicates that relational structure among occupations provides information that is not fully recoverable from sequential career history alone. In other words, the proposed Graphical Propagation Layer effectively supplements the sequence encoder with transition-relevant structural dependencies. The Dynamic Feedback Mechanism also makes a substantial contribution. Without it, performance falls from 0.398 to 0.381 in MRR and from 0.667 to 0.644 in Recall@10 on KARRIEREWEGE, with a similar trend on DECORTE, where MRR decreases from 0.371 to 0.355. These results suggest that the Dynamic Feedback Mechanism improves representation quality by enabling temporally adaptive updates during career trajectory modeling, which is especially useful for capturing evolving transition patterns. The Adaptive Career Pathway Strategy has a slightly different effect: removing it leads to a relatively stronger drop in top-ranked prediction quality, such as a decrease in Recall@1 from 0.249 to 0.234 on KARRIEREWEGE and from 0.229 to 0.214 on DECORTE. This supports the interpretation that the strategy improves pathway-level decision refinement and is particularly beneficial for ranking the most relevant target occupations at the top positions. The Encoder + Recommendation head only variant yields the weakest results among all ablated models on both datasets, confirming that the gains do not arise from the backbone architecture alone. Instead, the full improvement is achieved through the complementary interaction of structural propagation, dynamic temporal feedback, and adaptive pathway refinement. The relatively small standard deviations of the full model further indicate that these modules not only improve accuracy but also preserve stable optimization behavior across repeated runs.

**Table 4:** Module ablation results on KARRIEREWEGE and DECORTE.

Variant	MRR	Recall@1	Recall@5	Recall@10
KARRIEREWEGE				
Full model	0.398 ± 0.004	0.249 ± 0.004	0.531 ± 0.006	0.667 ± 0.006
w/o Graphical Propagation Layer	0.372 ± 0.005	0.228 ± 0.005	0.501 ± 0.007	0.632 ± 0.007
w/o Dynamic Feedback Mechanism	0.381 ± 0.005	0.236 ± 0.005	0.512 ± 0.006	0.644 ± 0.007
w/o Adaptive Career Pathway Strategy	0.384 ± 0.005	0.234 ± 0.005	0.516 ± 0.006	0.649 ± 0.006
Encoder + Recommendation head only	0.356 ± 0.006	0.217 ± 0.006	0.486 ± 0.008	0.618 ± 0.008
**DECORTE**				
Full model	0.371 ± 0.006	0.229 ± 0.006	0.506 ± 0.007	0.641 ± 0.008
w/o Graphical Propagation Layer	0.346 ± 0.007	0.209 ± 0.006	0.478 ± 0.008	0.608 ± 0.009
w/o Dynamic Feedback Mechanism	0.355 ± 0.007	0.216 ± 0.006	0.487 ± 0.008	0.619 ± 0.008
w/o Adaptive Career Pathway Strategy	0.358 ± 0.006	0.214 ± 0.006	0.491 ± 0.007	0.623 ± 0.008
Encoder + Recommendation head only	0.333 ± 0.008	0.198 ± 0.007	0.463 ± 0.009	0.596 ± 0.010

All values are reported as mean ± standard deviation over 10 independent runs.

Table 5 shows that the proposed framework maintains stable performance under moderate hyperparameter changes and degrades gracefully when historical input is intentionally restricted. On both KARRIEREWEGE and DECORTE, increasing the embedding dimension from 64 to 128 yields consistent but relatively modest improvements. For example, on KARRIEREWEGE, MRR increases from 0.389 to 0.398 and Recall@10 from 0.654 to 0.667, while on DECORTE, MRR increases from 0.362 to 0.371 and Recall@10 from 0.629 to 0.641. This indicates that finer representation granularity is beneficial, but the model does not rely on a narrowly tuned embedding size to achieve strong performance. A similar pattern appears for encoder depth. Using three encoder layers gives slightly better results than two layers on both datasets, but the gains remain controlled, suggesting that the proposed architecture is not overly sensitive to small depth changes and already captures most useful transition information at a moderate model capacity. The effect of maximum sequence length is also consistent with the task characteristics. Extending the sequence length from 10 to 20 improves all four metrics on both datasets, which suggests that preserving a longer occupational history helps the model recover additional transition context. However, the improvements are incremental rather than dramatic, implying that the framework can still perform competitively even when sequence context is moderately shortened. This behavior supports the claim that the model is robust to reasonable configuration changes rather than being tied to one specific setting. The robustness results under sparse history input are particularly important for practical career guidance scenarios. When the historical trajectory is restricted, performance decreases from 0.398 to 0.361 in MRR on KARRIEREWEGE and from 0.371 to 0.338 on DECORTE, with corresponding drops across all recall metrics. Nevertheless, the degradation remains gradual rather than severe, and Recall@10 still reaches 0.623 on KARRIEREWEGE and 0.601 on DECORTE. These results indicate that the proposed system can still produce useful predictions when user history is incomplete, which is a realistic and desirable property for real-world deployment. Overall, the table confirms that our method is both configuration-stable and robust to sparse career trajectories.

**Table 5:** Sensitivity and robustness analysis on KARRIEREWEGE and DECORTE.

Setting	MRR	Recall@1	Recall@5	Recall@10
KARRIEREWEGE				
Embedding dim = 64	0.389 ± 0.005	0.241 ± 0.005	0.521 ± 0.006	0.654 ± 0.007
Embedding dim = 128	0.398 ± 0.004	0.249 ± 0.004	0.531 ± 0.006	0.667 ± 0.006
Encoder layers = 2	0.391 ± 0.005	0.243 ± 0.005	0.523 ± 0.006	0.657 ± 0.007
Encoder layers = 3	0.398 ± 0.004	0.249 ± 0.004	0.531 ± 0.006	0.667 ± 0.006
Max sequence length = 10	0.386 ± 0.005	0.238 ± 0.005	0.517 ± 0.007	0.651 ± 0.007
Max sequence length = 20	0.398 ± 0.004	0.249 ± 0.004	0.531 ± 0.006	0.667 ± 0.006
Sparse history input	0.361 ± 0.006	0.220 ± 0.006	0.491 ± 0.008	0.623 ± 0.008
Full history input	0.398 ± 0.004	0.249 ± 0.004	0.531 ± 0.006	0.667 ± 0.006
**DECORTE**				
Embedding dim = 64	0.362 ± 0.007	0.221 ± 0.006	0.496 ± 0.008	0.629 ± 0.008
Embedding dim = 128	0.371 ± 0.006	0.229 ± 0.006	0.506 ± 0.007	0.641 ± 0.008
Encoder layers = 2	0.365 ± 0.007	0.224 ± 0.006	0.499 ± 0.008	0.633 ± 0.008
Encoder layers = 3	0.371 ± 0.006	0.229 ± 0.006	0.506 ± 0.007	0.641 ± 0.008
Max sequence length = 10	0.359 ± 0.007	0.219 ± 0.006	0.492 ± 0.008	0.626 ± 0.009
Max sequence length = 20	0.371 ± 0.006	0.229 ± 0.006	0.506 ± 0.007	0.641 ± 0.008
Sparse history input	0.338 ± 0.008	0.201 ± 0.007	0.468 ± 0.009	0.601 ± 0.010
Full history input	0.371 ± 0.006	0.229 ± 0.006	0.506 ± 0.007	0.641 ± 0.008

The sensitivity part uses three binary factors, and the robustness part evaluates sparse history inputs. All values are reported as mean ± standard deviation over 10 independent runs.

Beyond technical improvement, the observed performance gains also have important implications for psychology and education. The results suggest that student career development cannot be sufficiently characterized by simple historical repetition or static occupational similarity alone. Instead, more accurate prediction requires representations that capture developmental tendencies, differentiated transition patterns, and adaptive changes over time. From a psychological perspective, this supports the view that career development is a dynamic process shaped by multiple interacting factors, rather than a fixed one time matching problem. From an educational perspective, the findings indicate that more individualized and adaptive guidance frameworks may better support students' planning, reflection, and decision readiness in higher education contexts. The practical value of these improvements should also be considered in relation to how career guidance is actually used. In educational settings, guidance systems usually provide a short ranked list of plausible pathways rather than a single deterministic recommendation. Under such conditions, even moderate improvements in MRR, Recall@1, Recall@5, and Recall@10 may be meaningful because they increase the likelihood that relevant career options appear in the top positions presented to students and counselors. This may improve the usefulness of guidance conversations, make personalized support more timely, and help students explore pathways that are more consistent with their characteristics and developmental needs. Therefore, the significance of the proposed method lies not only in improved benchmark performance, but also in its potential to support more psychologically informed and educationally meaningful career guidance.

## 5 Discussion

From a theoretical perspective, the proposed framework can be interpreted in relation to established concepts in educational psychology and career development. The psychological profiling module corresponds to the assessment of individual differences, including personality traits, motivational orientation, and interest structures, which are widely recognized as important factors in learning behavior and career decision-making. Rather than treating these attributes as fixed labels, the model represents them as latent and developmental features that may influence students' engagement, preferences, and readiness for future planning. The multimodal encoder further integrates heterogeneous student information into a unified representation, which reflects the interaction of cognitive, affective, and experiential factors emphasized in educational psychology. The Adaptive Career Pathway Strategy can be understood in relation to dynamic perspectives of career development. Instead of viewing career choice as a one-time matching problem, the framework models guidance as an iterative and adaptive process, in which recommendations are continuously refined based on new information and feedback. This interpretation aligns with the view that career development involves ongoing adjustment, exploration, and re-evaluation, particularly in higher education contexts where students' interests, competencies, and aspirations evolve over time. In this sense, the proposed method reflects a shift from static recommendation toward development-oriented guidance. In practical educational and counseling contexts, the proposed framework is intended to function as a supportive decision tool rather than a deterministic system. The model can assist educators and counselors by organizing complex student information, identifying potential patterns, and generating a set of plausible career pathways for further discussion and reflection. However, career decisions remain influenced by contextual, social, and personal factors that are not fully captured by model inputs. Psychological assessments may also involve measurement error, self-report bias, and cultural limitations. Therefore, model outputs should be interpreted as supportive suggestions rather than authoritative judgments. The role of the framework is to enhance the informational and analytical basis of career guidance, while preserving the central role of human judgment, professional expertise, and student agency in decision-making.

## 6 Conclusions and future work

This study addressed the growing need for personalized career guidance in higher education by developing the Psychological Profiling-Driven Career Guidance Model (PPDCGM). The proposed framework connects psychologically informed modeling principles with machine learning techniques to provide precise and adaptive career recommendations tailored to individual student characteristics and developmental needs. The PPDCGM is further enhanced by the Adaptive Career Pathway Strategy, which enables dynamic adjustment of career guidance according to evolving learner states and external career conditions. Through benchmark experiments, efficiency comparisons, and ablation analyses, the proposed framework demonstrated effectiveness in improving career prediction quality, robustness, and effectiveness-efficiency trade-offs.

Despite these promising results, several limitations remain. First, although the framework is motivated by psychological profiling, the current benchmark-based evaluation relies primarily on observable career trajectory data rather than complete and standardized psychological assessment records. Future work should therefore investigate how richer psychological measurements can be incorporated into the framework under reliable and privacy-preserving data collection settings. Second, while the Adaptive Career Pathway Strategy shows clear potential, its practical value could be further strengthened by incorporating more timely external signals, such as labor market dynamics and industry demand changes. Future research may further extend the framework to more diverse educational settings, integrate additional multimodal data sources, and explore more realistic deployment scenarios for personalized career guidance. These directions may further improve the precision, adaptability, and real-world usefulness of the proposed system.

## Data Availability

The original contributions presented in the study are included in the article/supplementary material, further inquiries can be directed to the corresponding author.
